# Mental Capacity Act (Northern Ireland) 2016^†^

**DOI:** 10.1192/pb.bp.117.056945

**Published:** 2017-12

**Authors:** Gerard Lynch, Catherine Taggart, Philip Campbell

**Affiliations:** 1 Royal College of Psychiatrists in Northern Ireland; 2 Northern Health and Social Care Trust; 3 Belfast Health and Social Care Trust

## Abstract

Mental health legislation in Northern Ireland has always been separate from legislation in the rest of the UK; the Mental Health (Northern Ireland) Order (MHO) had been in place since 1986. In common with other jurisdictions, this legislation utilises the presence of mental disorder and risk as criteria for detention and involuntary treatment. The MHO has been replaced by the Mental Capacity Act (Northern Ireland) 2016 (MCA), an example of ‘fusion’ legislation in which impairment of decision-making capacity and best interests are the only criteria to be used when making decisions across health and social care. In this paper, we outline the development of the MCA to date, and discuss its potential to improve mental healthcare by placing the treatment of mental illness within the same legislative framework as physical illnesses.

The Mental Capacity Act (Northern Ireland) 2016 (MCA) is an example of a ‘fusion’ legislation – a generic law applicable across all medical specialties and social care where an intervention is proposed and the person has impaired decision-making capacity.

The MCA was enacted in May 2016. Since then, the Department of Health has completed phase 1 of the implementation work; creating a first working draft of a code of practice, and working on forms and draft regulations associated with the Act. This work has been shared with a ‘virtual’ MCA Reference Group, composed of a wide range of stakeholders. The first full draft of the code of practice, forms and regulations have recently been circulated to the MCA Reference Group and the second phase, a pre-consultation phase on the full draft, has just begun.

## Background

The Bamford review of mental health and learning disability, established in 2002, was a wide-ranging examination of the delivery of mental health and learning disability services in Northern Ireland.

In addition to the examination of service delivery, the review also undertook a review of the mental health legislation, the Mental Health (Northern Ireland) Order (MHO), which had been on the statute books since 1986. Criteria for involuntary treatment of mental illness under the MHO were based on diagnosis and risk; the presence of mental illness or severe mental impairment, and failure to detain leading to a substantial risk of serious physical harm to self or others. In addition, certain conditions were specifically excluded – no person could be detained solely on the grounds of personality disorder, dependence on alcohol or other drugs, or sexual deviancy.

There was no specific or separate mental capacity legislation in Northern Ireland. Decisions on the treatment of incapacitous patients are taken under common law, with decisions based on a presumption of capacity and the doctrine of necessity (best interests).

The Bamford review decided that the legislation was not compliant with essential principles (autonomy, justice, benefit and least harm). In 2007, it recommended that:^1^

- There should be a single comprehensive legislative framework for the reform of mental health legislation and the introduction of capacity.
- The framework should be based on agreed principles.
- These principles should apply to all healthcare decisions, as well as welfare and financial needs.
- Impairment of decision-making capacity should be a mandatory prerequisite for any interference with a person's autonomy without their consent.
- Individuals who are subject to the criminal justice system should have access to assessment, treatment and care which is equivalent to that available to other people.

A public consultation was held in 2011. There was strong support for the proposal of a single legislative framework. It was therefore decided to fuse mental capacity and mental health law into a single bill. The resulting draft bill was the subject of another consultation in 2014, followed by its introduction to the Stormont Assembly. There, it underwent further consideration and amendment, and was passed as the Mental Capacity Act (Northern Ireland), receiving Royal Assent in May 2016.

## Aims and principles

Fusion legislation provides equally for all circumstances in which a person's autonomy might be compromised on health grounds. It puts impaired decision-making capacity at the heart of all non-consensual interventions. By treating mental and physical illnesses equally under the law, it reduces stigma associated with separate mental health legislation, and is respectful of a person's autonomy and decision-making capacity whether they have a mental or a physical illness.

Compatibility with international statements on human rights, particularly the European Convention on Human Rights (ECHR)^2^ and the United Nation's Convention on the Rights of Persons with Disabilities (CRPD),^3^ is an issue for any legislation dealing with involuntary treatment. Conventional mental health legislation, which uses a diagnostic test for involuntary treatment, could be regarded as being incompatible with Article 14 (1) (b) of the CRPD – ‘the existence of a disability shall in no case justify a deprivation of liberty’. No UK mental health legislation is currently compliant with the CRPD.

Proponents of fusion legislation argue that capacity, as the test for involuntary treatment, is a functional test, i.e. a particular ability at a particular time, and therefore not directly linked to diagnosis or disability. However, the MCA retains a ‘diagnostic’ element; the person is unable to make a decision because of an impairment of, or a disturbance in, the functioning of the mind or brain. It therefore arguably still fails to satisfy Article 14 (1) (b) of the CRPD. Nevertheless, the MCA is fully compliant with the ECHR, and its strong rights- and principles-based ethos moves the legislation significantly towards CRPD compliance.

## Content of the MCA

The MCA revokes the MHO for those aged 16 and over and puts common law into statute.

### Capacity test

Statutory decision-making will come into play when a person lacks capacity. There is a presumption that the person has capacity, there must be no unjustified assumptions based on age, appearance or condition, there must be a respect for decisions even if unwise, the person must be given all practical help and support, and the act must always be in the person's best interests.

There are two tests to be satisfied in reaching a decision about a person's decision-making capacity:

1. a diagnostic test – there must be an impairment of, or a disturbance in, the functioning of the mind or brain, and;
2. a functional test – the person is unable to understand the information relevant to the decision, to retain the information long enough to make the decision, to appreciate the relevance of that information and use or weigh the information as part of the process of making that decision, and communicate the decision.

There must be a causal link between the two tests – the person is unable to make a decision because of impairment or disturbance in the brain or mind.

Looking at the functional test, the specific difference between this and other definitions of lack of capacity is the use of the word ‘appreciate’. The consultation document^4^ emphasises the importance of the inclusion of the appreciation element: its inclusion moves a decision about capacity from a purely cognitive test (p. 13, para. 2.22). The consultation document gives as an example: ‘A person whose insight is distorted by their illness or a person suffering from delusional thinking as a result of their illness may not, therefore, meet this element of the test’ (p. 13, para. 2.22).^4^

### Protection from liability

The legislation puts into statute the common-law definition of necessity and protects the person (D) doing the act from liability if D takes reasonable steps to establish whether the person (P) lacks capacity in relation to the matter in question and D reasonably believes that it is in P's best interests for the act to be done. There is therefore a shift in emphasis from the MHO, which confers statutory powers, to a situation where non-consensual intervention is predicated on protection from liability for D.

### Future decision-making

The Act includes a robust lasting powers of attorney system. A lasting powers of attorney must be registered with the Office of Public Guardian before being activated and extends to health and welfare decisions, when the attorney reasonable believes that the person lacks capacity and must always act in the person's best interests.

In addition, advance decisions to refuse treatment must be complied with, if valid and applicable under common law. This means that an effective advance decision to refuse treatment for a mental disorder (or indeed any disorder) cannot be overridden, if made when P had capacity. However, the Act allows that, if there is doubt, D will be protected from liability if he or she gives life-sustaining treatment or treatment required to prevent a serious deterioration in P's condition. Advance decisions were not put into statute in order for the courts to continue to develop the law in the light of the MCA.

### Safeguards

The Act provides for a proportionate increase in the number of safeguards that must be met if D is to be protected from liability as the seriousness of the interventions or acts being done to P increases. These additional safeguards must be met in addition to the general safeguards.

- For acts of restraint, D must reasonably believe that there is a risk of harm to P, and that the act of restraint is proportionate to the likelihood and seriousness of that harm.
- For serious interventions or treatment with serious consequences, there must be a formal assessment of capacity and a written statement of incapacity by a suitably qualified person, and a nominated person must be in place, who should be consulted and whose views should be taken into account. Serious interventions include, but are not limited to, serious treatment for physical illness, any intervention that causes the person serious distress or serious side-effects, affects seriously the options that will be available to P in the future or has a serious effect on his/her day-to-day life. The decision whether or not an act is a serious intervention or treatment with serious consequences rests with D. However, some acts are always serious interventions. These are: (a) deprivation of liberty, (b) attendance for certain treatments requirement and (c) community residence requirement.
- Certain serious interventions must be authorised by a trust panel. These include acts (a), (b) and (c) above, or the act is the provision of treatment with serious consequences and the nominated person objects, P resists or it is being done while the person is being deprived of their liberty.
- For attendance for certain treatment requirements, D must reasonably believe that failure to impose the requirement would be more likely than not to result in P not receiving the treatment.
- For community residence requirements, the prevention of harm condition must be met.

The trust panel will be made up of three persons with relevant expertise. The application will be made by a ‘prescribed person’ and must include a medical report and a care plan. The statutory criteria will differ depending on the measure for which authorisation is being sought.

- For treatment with serious consequences when the act amounts to a deprivation of liberty, the ‘prevention of serious harm’ condition must be met. D must reasonably believe that failure to detain P in circumstances amounting to a deprivation of liberty would create a risk of serious harm to P or serious physical harm to others, and the detention of P is a proportionate response to the likelihood of harm and the seriousness of the harm concerned.
- For attendance for certain treatment requirements, D must reasonably believe that failure to impose the requirement would be more likely than not to result in the person not receiving the treatment.
- For community residence requirements, the ‘prevention of harm’ condition must be met.
- For compulsory treatment with serious consequences against the wishes of the nominated person, the ‘prevention of serious harm’ condition must be met.

A second opinion is required when the act is the provision of electroconvulsive therapy or is a treatment with serious consequences where the question of best interests is finely balanced, or is the continuation of medication beyond 3 months (if the medication is treatment with serious consequences) when the person is an in-patient or in a care home, or is subject to requirements to attend for treatment in the community.

The Act provides for the provision of an independent mental capacity advocate (IMCA). An IMCA must be in place when the Act requires an act to be authorised or, although not requiring authorisation, is a serious compulsory intervention. The role of the IMCA is to support and represent P; the IMCA must be consulted but is not a decision maker.

Where an authorisation has been granted, an application can be made to a review tribunal in respect of the authorisation. This provides a judicial review of the decision to ensure that it has been made in accordance with the law and that the criteria for the authorisation have been met. Applications to the tribunal can be made by P and the nominated person. Cases may also be referred to the tribunal by the Department of Health, the Attorney General or the High Court. The trust must refer to tribunals when authorisation has been extended for 1 year (for those aged 16–17) or 2 years (for those aged 18 or over).

The clauses describing the additional safeguards to be put in place do not apply when the situation is an emergency. D is protected from liability if there is a reasonable belief that delay would create an unacceptable risk of harm to P. However, D is expected to take reasonable steps to ensure that the safeguard is met by the relevant time.

## Children and young people

The Act cannot be applied to children under the age of 16 because it puts into statute the common law presumption of capacity. For those aged 16–17, the MCA will operate alongside the Children (Northern Ireland) Order 1995, and additional safeguards will be put in place. The original MHO will continue to be in place for the small number of under-16s who require compulsory assessment/treatment in hospital for mental disorder. This has been the subject of some controversy; if the current legislation is discriminatory and stigmatising, it is difficult to argue for its continued use in one particular group. It has been argued that a legislative framework for those under 16 must be brought forward. This will be a difficult task, not least because a capacity-based framework will have to grapple with the complex question of emerging capacity in young people. The government has indicated that their intention is that there will eventually be legislation for those under 16, but at present, this is some way off.

## Criminal justice provisions

There are new disposal options following a finding of unfitness to plead or insanity, including public protection orders (PPOs) and supervision and assessment orders. There are powers to remand an accused person to hospital, to transfer prisoners to hospital for treatment, for interim detention orders and for immediate hospital direction on conviction. Although the MCA contains powers for involuntary admission to hospital in various circumstances, treatment decisions are based on capacity to consent and subject to the core provisions of the Act. This means that there are circumstances under which a person can be admitted to hospital against their capacitous wishes; however, they cannot be treated against their capacitous wishes.

New criteria form the basis for entry into the criminal justice provisions. A ‘disorder’, a ‘disorder requiring treatment’ and ‘an impairment of, or disturbance in, the functioning of the offender's mind or brain’ replace mental illness and severe mental impairment. A disorder is broadly defined to include any disorder or disability, whether mental or physical: a disorder requires treatment if any of its symptoms or manifestations could be alleviated or prevented from worsening by treatment.

A person can be remanded to hospital if the medical report condition or the treatment condition are met. The medical report condition is that the person has or may have a disorder, that a report should be made into that person's condition, that an assessment would be impracticable in custody, and that it would be practicable to assess the person in hospital. The treatment condition is that the person has a disorder requiring treatment, that failure to provide in-patient treatment would ‘more likely than not’ result in serious physical or psychological harm to the accused person or serious physical harm to others, and that remanding the person to hospital would be likely to result in significantly better clinical outcomes.

PPOs replace hospital orders. A PPO can be made when detention conditions are met. These are that: ‘there is an impairment of, or a disturbance in, the offender's mind or brain’, that ‘appropriate care and treatment is available’, that dealing with the person without detention ‘would create a risk, linked to the impairment or disturbance, of serious physical or psychological harm to others’ and that depriving the person of their liberty would be a proportionate response to the likelihood and seriousness of that harm. Restrictions may be added where the restriction conditions are met.

A prisoner can be transferred to hospital where they have a disorder requiring treatment, failure to provide treatment would be ‘more likely than not’ to result in serious harm to the person or serious physical harm to others, and appropriate treatment is available.

Patients admitted to hospital under the MCA criminal justice provisions will remain there following tribunal only if the ‘prevention of serious harm’ condition is met. The criteria for the ‘prevention of serious harm’ condition differ for those subject to PPO and for transferred prisoners or those subject to hospital direction. The criteria for those subject to PPO are:

- the person has ‘an impairment of, or a disturbance in, the functioning of the mind or brain’;
- releasing the person would create a risk of serious harm to others; and
- depriving the person of their liberty is proportionate to the likelihood and seriousness of the risk.

The criteria for transferred prisoners or those subject to hospital direction are:

- the person has the disorder for which they were transferred;
- effective treatment can be given; and
- it is ‘more likely than not’ that discharging the person to prison would result in serious harm to the person or serious physical harm to others.

## Discussion

The MCA is unique in that it repeals separate mental health legislation, replacing it with a single piece of legislation applicable across all medical specialisms and social care, whereby involuntary treatment is only permitted when the person (a) has impairment of decision-making capacity and (b) the intervention proposed is in the person's best interests.

The arguments for and against replacing conventional mental health legislation with a law based on capacity have been well rehearsed in a recent debate.^5^

The removal of mental health legislation that makes decisions about involuntary treatment based on diagnosis and risk will require a significant change in practice for professionals working in mental health in Northern Ireland. It is somewhat ironic that such a radical piece of legislation, based on non-discrimination, is being introduced in a jurisdiction that spends the lowest proportion of its health budget on mental health of any UK nation.^6^

The Act must work across a wide and diverse range of settings – care homes, mental health services (both in-patient and community) and general hospitals. It will affect staff who have little previous knowledge or experience of the principles behind capacity assessment. It is therefore imperative that a comprehensive training and supervision programme is put in place, which will have considerable resource implications.

The inclusion of the ‘appreciation’ element introduces a difference in the definition of capacity in the MCA compared with that used in other jurisdictions. The addition of the ‘appreciation’ element moves the definition of capacity away from purely cognitive terms towards the concept of capacity being affected by factors such as emotional colouring, delusions and lack of insight.^7^ Because of this difference, it cannot be assumed that studies that have demonstrated the reliability of capacity assessments^8^ will automatically apply in the case of the MCA. The reliability of the use of capacity assessments using this definition of capacity in routine clinical mental health practice requires to be evaluated.

The shift away from compulsory intervention based on in-patient treatment when a particular threshold of risk is reached may facilitate earlier intervention and allow for a proportionate response across a wide range of treatment and care settings. On the other hand, there is a more widely expressed concern that capacity legislation may delay appropriate treatment.

Trust panels can authorise a very wide range of interventions. As health and social care professionals work under the principle of beneficence, there is a risk of ‘slippage’, with staff making decisions about impaired capacity based on a person making foolish or unwise choices. This could lead to the unintended consequence of the Act leading to a greater rather than a lesser restriction of a person's autonomy and self-determination.

There is a plethora of issues that could potentially affect clinical practice; for example, exactly what constitutes serious interventions, how to manage fluctuating capacity, the question of decision-making capacity in patients with personality disorder, patients who retain capacity but present a risk to self or others, and the potential conflict between human rights (especially the right to life) and autonomy. Some of these issues may be addressed by the code of practice, others may be left to clinicians or courts.

## Conclusions

Fusion legislation (of which the MCA is an example) is a radical change in the approach to involuntary psychiatric treatment. It is an exciting and innovative development and there are substantial potential benefits, including the reduction of stigma, the protection of patient autonomy, and the removal of confusing parallel mental health and mental capacity legislation. It is also more compliant with CRPD and ECHR. Much of the practical impact of the MCA depends on the development of a comprehensive code of practice and the provision of a comprehensive training and supervision programme. In addition, as Szmukler & Kelly have pointed out,^5^ the gathering of data on its implementation is vital and the MCA must be subject to a rigorous and comprehensive evaluation.
